# Practical Notes on Diagnosis and Treatment

**Published:** 1910-05-14

**Authors:** 


### PRACTICAL NOTES ON DIAGNOSIS AND TREATMENT.

**Stammering.**

This habit is more frequently imitated than inherited.—*Dr. L. Guthrie.*

**Erythema Multiforme.**

A long course of arsenic is useful in preventing the recurrence of attacks of erythema multiforme.—*Mr. Jonathan Hutchinson.*

**The Mammæ and Phthisis.**

In early cases in female patients there is often to be noted a marked decrease in the size of the breast on the affected side.—*Dr. Richter.*

**Pain in Osteo-arthritis.**

Ichthyol may give great relief when applied externally. One part of ichthyol to two parts of olive oil is generally of sufficient strength.

**Anæmia and Phthisis.**

The early detection of tubercular mischief in anæmic subjects is of vital importance, for, in my experience, the disease in such persons tends to advance rapidly.—*Dr. J. E. Squire.*

**Fungating Endocarditis.**

Spontaneous hæmorrhages in a heart case are very suggestive. It is rare for simple valvular heart disease to cause hæmorrhages other than hæmoptysis or menorrhagia. Should petechiæ appear under the skin or blood in the urine, a suspicion of fungating endocarditis at once arises.

**Reflexes in Hysteria.**

The knee-jerks are never absent on reinforcement. True ankle clonus is never obtained in hysteria, and if the plantar reflex is obtained the toes never go upwards. The pharyngeal reflex is frequently abolished, so that the finger may feel the glottis without obvious discomfort to the patient. On the other hand, the corneal reflex is rarely absent. One of the most characteristic features of hysterical anæthesia is the fact that the superficial reflexes can usually be obtained from parts that are completely anæsthetic.

**Addison's Disease.**

If at the end of two or three years after a diagnosis of Addison's disease has been made the patient is still alive, revise the diagnosis.—*Dr. Hale White.*

**Nasal Polypi.**

These growths are associated with disease of the underlying bone—a rarefying osteitis; hence the speedy recurrence of polypi after snare operations, a method which is but a temporary palliation in extensive cases.

**Tumours of the Breast.**

It is dangerous to regard any tumour of the breast as innocent. In a series of 162 cases examined microscopically more than four out of five proved to be malignant. An exploratory incision should be made before expressing any opinion as to the innocent nature of a tumour.

**Whooping Cough.**

This disease is to be regarded as a neurosis superimposed on to a lesion of the respiratory mucous membrane, and the neurosis is really the more important part of the affection. Suggestion plays a large part in aggravating the cough in children sufferering from mild attacks. Isolation in bed is an important part of the treatment, which should be directed principally to the nervous system.—*Professor Czerny.*

**Reflexes in Scarlet Fever.**

In many cases of scarlet fever the normal plantar response of flexion is temporarily replaced by extension. The severer the case, and the younger the patient, the more likely is this change to occur, and, generally speaking, the severer the case the longer the duration of this phenomenon. The knee-jerks were rarely found to be absent even in severe cases. The cremasteric reflex is the briskest in early youth, and is scarcely ever abolished. Subjects attacked with endocarditis showed more derangement of their reflexes than those afflicted with other complications of scarlet fever.—*Dr. C. Rolleston.*

